# Macronutrient application rescues performance of tolerant sorghum genotypes when infected by the parasitic plant striga

**DOI:** 10.1093/aob/mcae031

**Published:** 2024-03-01

**Authors:** Immaculate M Mwangangi, Lucie Büchi, Stephan M Haefele, Jonne Rodenburg

**Affiliations:** Natural Resources Institute, University of Greenwich, Chatham Maritime, Kent, ME4 4TB, UK; Natural Resources Institute, University of Greenwich, Chatham Maritime, Kent, ME4 4TB, UK; Sustainable Soils and Crops Department, Rothamsted Research, Harpenden, Hertfordshire, AL5 2JQ, UK; Natural Resources Institute, University of Greenwich, Chatham Maritime, Kent, ME4 4TB, UK

**Keywords:** Host tolerance, root parasitic weed, *Sorghum bicolor*, *Striga hermonthica*, witchweed

## Abstract

**Background and Aims:**

Infection by the hemi-parasitic plant *Striga hermonthica* causes severe host plant damage and seed production losses. Increased availability of essential plant nutrients reduces infection. Whether, how and to what extent it also reduces striga-induced host plant damage has not been well studied.

**Methods:**

The effects of improved macro- and micronutrient supply on host plant performance under striga-free and infected conditions were investigated in glasshouse pot assays. One striga-sensitive and two striga-tolerant genotypes were compared. Plants growing in impoverished soils were supplied with (1) 25 % of optimal macro- and micronutrient quantities, (2) 25 % macro- and 100 % micronutrients, (3) 100 % macro- and 25 % micronutrients, or (4) 100 % macro- and micronutrients.

**Key Results:**

Photosynthesis rates of striga-infected plants of the sensitive genotype increased with improved nutrition (from 12.2 to 22.1 μmol m^−2^ s^−1^) but remained below striga-free levels (34.9–38.8 μmol m^−2^ s^−1^). For the tolerant genotypes, increased macronutrient supply offset striga-induced photosynthesis losses. Striga-induced relative grain losses of 100 % for the sensitive genotype were reduced to 74 % by increased macronutrients. Grain losses of 80 % in the tolerant Ochuti genotype, incurred at low nutrient supply, were reduced to 5 % by improved nutrient supply.

**Conclusions:**

Increasing macronutrient supply reduces the impact of striga on host plants but can only restore losses when applied to genotypes with a tolerant background.

## INTRODUCTION

The parasitic plant *Striga hermonthica* [Del.] Benth. is endemic to sub-Saharan Africa. As a weed in field-grown cereal crops (e.g. sorghum, maize, millet and rice), it causes severe yield losses with resulting high impacts on regional food security and the economy (Rodenburg *et al.*, 2006; Waddington *et al.*, 2010). *Striga hermonthica* (hereafter striga) belongs to the family Orobanchaceae and is an obligate hemi-parasite that depends on a monocotyledon host for germination and growth (Spallek *et al.*, 2013). Striga germinates following perception of host plant-derived hormones (such as strigolactones) present in the rhizosphere (Yoneyama *et al.*, 2007) and forms a root penetration structure called haustorium to establish a vascular connection with the host root (Saunders, 1933; Williams, 1961; Riopel and Baird, 1987). Once established, striga extracts water, carbon assimilates and amino acids from the host plant (Hood *et al.*, 1997; Yoshida *et al.*, 2016). Attached striga plants also transfer phytotoxic secondary metabolites (iridoid glycosides) to the host, but their role is poorly understood (Rank *et al.*, 2004). The parasite further excretes abscisic acid (ABA) around the host root rhizosphere that suppresses the host immune system and growth performance (Frost *et al.*, 1997; Fujioka *et al.*, 2019), reduces the host leaf stomatal conductance rate and affects the host photosynthesis apparatus (Frost *et al.*, 1997). The latter two effects have a pronounced negative impact on the CO_2_ assimilation rate of the host plant (Graves *et al.*, 1988; Gurney *et al.*, 1995, 2002; Rodenburg *et al.*, 2008, 2017). The cumulative pathogenic effect of *Striga* spp. on the host plants stunts growth, increases the root : shoot ratio and induces drought-like symptoms, resulting in significant grain production losses (Gurney *et al.*, 1995, 1999; Dörr, 1997; Rank *et al.*, 2004). At the crop level, yield losses caused by *Striga* spp. infection range from 21 to 80 % in rice (Rodenburg *et al.*, 2016), from 10 to 81 % in maize (Kim *et al.*, 2002) and from 0 to 84 % in sorghum (Rodenburg *et al.*, 2005), depending on striga seed density in the soil and the effectiveness of the striga defence mechanisms of the host plant. The latter is genotype-specific and various host genotypes of different crop species have been identified with increased levels of striga resistance or tolerance (e.g. Omoigui *et al.*, 2017; Rodenburg *et al.*, 2017; Mallu *et al.*, 2021; Stanley *et al.*, 2021). The term resistance in this context refers to host plant traits that contribute to reduced striga infection levels and is always partial. Striga tolerance refers to physiological traits that enable the host plant to better withstand striga pathogenicity (Rodenburg and Bastiaans, 2011). The presence of physiological tolerance can be detected by reduced losses in CO_2_ assimilation rate and yields under striga-infected conditions, relative to other genotypes (Gurney *et al.*, 2002; Rodenburg *et al.*, 2008). Like striga resistance, the trait tolerance is unlikely to provide complete protection.

Nutrient availability also plays a crucial role in striga infection and effects. Under conditions of (macro-) nutrient deficiency, host plant roots release more strigolactones, acting as cues for striga germination (Yoneyama *et al.*, 2007). Conversely, several studies have shown the application of fertilizers containing macronutrients N and P reduces striga infection levels at pre-germination stages (Cechin and Press, 1993*a*; Jamil *et al.*, 2013, 2014). More recently, macronutrients have been observed to also reduce striga infection at the post-germination growth stages (Kokla *et al.*, 2022; Mwangangi *et al.*, 2023). Moreover, application of nitrogen fertilizer may reduce striga effects on the host plant. For example, the application of optimum levels of nitrogen on striga-infected pearl millet increased the host transpiration rate and, thereby, decreased the transfer of host nutrients to the parasite (Boukar *et al.*, 1996). Foliar application of nitrogen fertilizer to striga-infected host plants decreases the heterotrophic carbon dependency of the parasite on the host, enabling the host plant to maintain more assimilated carbon for its own use (Cechin and Press, 1993*b*).

So far, studies on the effect of nutrition on striga-infected host plants have focused only on nitrogen and phosphorus, and thereby potentially overlooked the impact of other essential elements. Despite the importance of other macronutrient elements such as magnesium, sulphur and calcium, as well as micronutrients such as boron, molybdenum, iron, zinc, copper and sodium in influencing host defence mechanisms (Mwangangi *et al.*, 2021), their specific effects on striga tolerance have not yet been investigated. Furthermore, the extent to which striga effects on the host plant performance can be reduced by improved nutrition, and whether this depends on the tolerance of the host plant to striga infection, has not been quantified.

The combination of nitrogen fertilizer and the use of tolerant genotypes has been proposed and studied previously (e.g. Kim *et al.*, 1997; Showemimo *et al.*, 2002), but in these studies the role of tolerance could not be disentangled from that of the resistance and inherent yield potential of the crop genotypes under review. This is explained by Rodenburg and Bastiaans (2011). Briefly, the level of tolerance in any crop genotype is measured by the extent to which plants of this genotype are affected by striga infection. To assess that, striga-infected plants of that genotype would need to be compared to striga-free plants of that same genotype. In the past, only very few field studies have included striga-free control plots to enable such assessment. Another hurdle to overcome, for a fair comparison of tolerance across genotypes, is the commonly observed difference in striga infection levels between genotypes in any experimental set-up, resulting from their inherent differences in striga resistance (Rodenburg *et al.*, 2005, 2006). This can only be overcome when genotypes are screened against a wide range of striga infestation levels, to obtain sufficient overlap in infection levels, or if only genotypes with similar resistance levels are compared. Partly due to the above outlined complications in the accurate assessment of striga tolerance, very few studies have focused on this trait and none have quantitatively determined the effect of fertilizers on striga tolerance before. Based on the drought-like symptoms that striga-infected host plants exhibit, nutrients that play a role in alleviating drought stress-induced effects might also improve striga tolerance (Hu *et al.*, 2018). Additionally, nutrients that play pivotal roles in host plant photosynthesis pathways could potentially mitigate the host plant effects due to striga infection, as such effects are characterized by impaired photosynthesis (Mwangangi *et al.*, 2021).

Therefore, the research questions addressed by the current study were: (1) to what extent does improved micro- or macronutrient supply play a role in mitigating the impact/effect of striga on host plant performance? and (2) does the extent of this reduction depend on the physiological tolerance level of the host plant?

## MATERIALS AND METHODS

### Experimental design and growth conditions

Three experiments were run between 2020 and 2022 in a split–split plot design with three replicates for experiment 1 and four replicates for experiments 2 and 3. The treatments included three sorghum genotypes (CK60B, Ochuti and Tiemarifing), nutrient application at four levels (Base, +Micro, +Macro, +MicroMacro, see below for explanations) and striga infestation at two levels (striga-infested and striga-free soil). The sorghum genotypes were allocated to the main plots, striga treatment to the sub plots and nutrient treatment to the sub-sub plots.

The three sorghum genotypes CK60B, Ochuti and Tiemarifing were selected based on differences in striga tolerance and comparable striga resistance levels (Table 1). Seeds of sorghum genotypes CK60B and Tiemarifing were obtained from Wageningen University (Netherlands) while seeds of Ochuti were acquired from Kenyatta University (Kenya). *Striga hermonthica* seeds were obtained from striga-infested sorghum farms in Alupe, western Kenya, in 2019.

**Table 1.** Name, race, origin and reported striga resistance and tolerance of the sorghum genotypes.

Genotype	Race	Origin	Resistance	Tolerance	Source
CK60B	Kafir	NE Africa/USA	Moderately susceptible	Sensitive	Rodenburg *et al.*, 2008
Ochuti	Durra	Kenya	Moderately susceptible	Tolerant	Gurney *et al.*, 1995
Tiemarifing	Guinea	Mali	Moderately susceptible	Tolerant	Rodenburg *et al.*, 2008

The nutrient treatments were calculated based on the Long Ashton composition (Hudson, 1967), with the quantity of each element calculated proportionally to the recommended nitrogen rate for field-grown sorghum (60 kg N ha^−1^, according to CABI and assuming a crop density of 40 000 plants ha^−1^). The salts used for these nutrient treatments were dissolved in water for ease of application (formulas and concentrations in Table 2). The nutrient treatments comprised four levels: (1) a low nutrient level (Base), consisting of 25 % of the optimal quantity of micro- and macronutrients; (2) an optimal micronutrient level (+Micro), with 100 % of micronutrients but a baseline of 25 % macronutrients; (3) an optimal macronutrient level (+Macro), with 100 % of macronutrients but 25 % of micronutrients; and (4) an optimal nutrient level (+MicroMacro) with 100 % of the required micro- and macronutrients. A total of 2.5 L of treatment-specific nutrient solutions were soil-applied to each plant in three splits, with 40 % of the total quantity applied at 10 d after sowing (DAS), another 40 % at 46 DAS and the last 20 % at 82 DAS. These application dates covered the vegetative stage (before 46 DAS), booting stage (between 46 and 82 DAS) and flowering (from 82 DAS onwards).

**Table 2.** Nutrient treatment composition based on the Long Ashton solution with a recommended nitrogen rate at field level (60 kg N ha^−1^; based on a crop density of 40 000 plants ha^−1^) as benchmark. Values represent the concentrations of the element in solution (mg L^−1^); a total of 2.5 L of this fertilizer solution was applied to each plant in three splits, at 10 d (40 %), 46 d (40 %) and 82 d (20 %) after sowing (DAS).

		Base	+Micro	+Macro	+MicroMacro
		25 % micro + 25 % macro	100 % micro + 25 % macro	25 % micro + 100 % macro	100 % micro + 100 % macro
NH_4_NO_3_	N	149.94	149.94	599.8	599.8
K_2_SO_4_	K	139.23	139.23	556.92	556.92
CaCl_2_.2H_2_O	Ca	142.8	142.8	571.2	571.2
Na_2_HPO_4_	P	36.6	36.6	146.4	146.4
MgSO_4_.7H_2_O	Mg	32.13	32.13	128.52	128.52
NaCl	Na	27.7	110.7	27.7	110.7
H_3_BO_3_	B	0.5	1.93	0.5	1.93
Fe (III)-EDTA	Fe	5.0	20.0	5.0	20.0
MnSO_4_.4H_2_O	Mn	0.5	2.0	0.5	2.0
ZnSO_4_.7H_2_O	Zn	0.1	0.23	0.1	0.23
CuSO_4_.5H_2_O	Cu	0.1	0.23	0.1	0.23
Na_2_MoO_4_.H_2_O	Mo	0.04	0.2	0.04	0.2

All three experiments were carried out in a glasshouse with supplemented LED light providing a mean estimated light intensity at canopy level of 386 μmol m^−2^ s^−1^. Experiment 1 was carried out from July (2020) to January (2021) at Rothamsted Research (UK) in Harpenden. Experiments 2 and 3 were carried out from May to September (2021) and March to July (2022), respectively, at the University of Greenwich, Medway campus (UK). Glasshouse conditions of experiments 2 and 3 were set at a minimum day temperature of 28 °C and a minimum night temperature of 21 °C, with day (light) length set at 12 h, using programmed black-out curtains, and 60 % relative humidity across the three experiments. For experiment 1 the only environmental conditions that were controlled were (minimum) temperature (21 °C) and light intensity.

For each experiment, 10-L pots (height: 20 cm; diameter: 23 cm) were filled with sand (Horticultural sharp sand, Melcourt, UK) and soil (Meadowmat low fertility soil, Harrowden Turf Limited, UK) in a ratio of 1 : 1. Half of the pots were infested with *S. hermonthica*, by mixing the upper 10-cm soil layer with striga seeds, following methods outlined by Rodenburg *et al.* (2008). For experiments 1 and 2, the top soil layer in each pot was mixed with 93.48 mg of striga seeds (implying 3 striga seeds cm^−3^), whereas for experiment 3 the infestation level was doubled to compensate for the decreased striga viability observed in experiments 1 and 2. The other half of each experiment was maintained striga-free.

The striga seeds were preconditioned for 7 d in the glasshouse by watering the soil in the pots daily to achieve a stable soil moisture content around the field capacity level (Rodenburg *et al.*, 2008; Mbuvi *et al.*, 2017). Pots with striga-free soils were treated the same. On the eighth day after striga preconditioning, three sorghum seeds were sown in the centre of each pot at 2–3 cm depth. Sorghum plants were then thinned at 7 d after sowing to maintain one host plant per pot. The sorghum plants were watered every day.

### Measurements and observations

The number of emerged striga plants was assessed from 28 to 88 DAS for all experiments at 7-d intervals for experiment 1 and every second day for experiments 2 and 3. The area under the striga number progress curve (ASNPC) was calculated based on these counts, as the sum of daily striga numbers or estimates (Haussmann *et al.*, 2000). Total above-ground striga dry biomass was assessed by collecting dead plants during the experiments and all remaining plants at harvest time. Striga plants were dried for 48 h at 70 °C and then weighed to determine dry weight.

Five non-destructive measurements of the host plant were recorded: leaf chlorophyll content, leaf photosynthesis and related stomatal conductance and electron transport (all at 60 DAS), and plant height (at 85 DAS). Leaf chlorophyll recorded per plant was the average value of six measurements halfway along the length of the youngest fully developed leaf, using a SPAD-502 chlorophyll meter (Konica Minolta, Tokyo, Japan).

Gas exchange rates were also measured halfway along the length of the youngest fully developed leaf using a portable photosynthesis measurement system (Li-6400XT for experiment 1, and Li-6800XT for experiments 2 and 3, LI-COR Bioscience, Lincoln, NE, USA). The net CO_2_ assimilation rate (*A*), stomatal conductance rate and electron transport rate through photosystem II (ETR) were used as leaf-level performance indicators in all the experiments following previous studies of striga effects on host plant physiology (e.g. Gurney *et al.*, 2002; Rodenburg *et al.*, 2008). Measurements were made at a CO_2_ concentration of 400 µmol mol^−1^, flow set point of 500 µmol s^−1^, 60 % relative humidity, leaf chamber temperature at 28 °C and light intensity of 2000 µmol m^−2^ s^−1^ (photosynthetically active radiation).

Host plant height was measured from the base of the stem to the ligule of the youngest fully developed leaf. Host plant height is a reliable indicator of striga effects (Press and Steward, 1987).

At maturity (around 120 DAS), the sorghum leaves and stems were dried at 70 °C for 48 h and weighed to determine the total dry biomass. The sorghum panicles were cut, air-dried and threshed, and the grains were weighed to determine the yield of each plant. The genotype Tiemarifing did not produce panicles and grains within the duration of the experiments, and therefore these data were only obtained for Ochuti and CK60B.

### Data analysis

For plant-level sorghum variables (height, biomass and yield), a relative loss (RL) ratio was calculated as the difference between a given variable (*vx*) measured on striga-free (C) and on striga-infected (S) plants, divided by the striga-free plant values, hence: RL_*vx*_ = (C_*vx*_ − S_*vx*_)/C_*vx*_, using averages across treatments. The RL of each variable was used to determine the effect of the nutrient treatments on the sorghum genotype response to striga infection. A relative measure was used to enable comparisons between sorghum genotypes which differed widely in inherent plant morphological traits. For leaf-level performance indicators (chlorophyll and gas exchange variables), the absolute values of the striga-free and striga-infected plants were used for analysis. These were previously demonstrated to be relatively constant and comparable across healthy plants of different genotypes (Rodenburg *et al.*, 2008).

A linear mixed model was used to test the effect of treatments (in a split-plot design with genotypes at the main plot level and nutrients at the sub-plot level) on the response variables measured using the R package ‘nlme’ (Pinheiro *et al.*, 2012). The three experiments were analysed together, with experimental runs and replicates within an experiment as a random factor in the models. Nutrient and sorghum genotype treatments were categorized as fixed effects for analyses of the relative plant-level variables, whereas nutrient, genotype and striga treatments were treated as fixed effects for analysis of absolute values of the leaf-level measurements. Based on the estimated marginal means from the mixed model, a Tukey honest significant difference (HSD) test was done to compare treatment means, using the ‘emmeans’ package (Russel, 2021). All data analyses were done using R 4.2.1 (R Core Team, 2021) and R studio 1.3.1073 (RStudio Team, 2021).

## RESULTS

### Striga infection

**Fig. 1.**
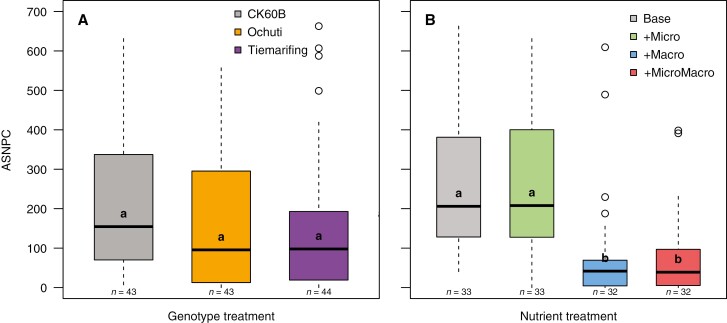
The effect of sorghum genotype across all nutrient treatments (A) and the effect of nutrient (B) treatments across all sorghum genotypes on the number of emerged striga plants. For both panels, boxes show the distribution of the data across all levels of the other factor (genotypes for A and nutrient treatments for B) across the three experiments. Boxes with different letters indicate significant differences at *P* < 0.05 based on a Tukey post-hoc comparison of estimated marginal means. Numbers under each box show the sample size. Bold horizontal lines in the boxes indicate the median value; boxes represent the upper and lower quartiles. Whiskers extend to the most extreme data points within ±1.5× the interquartile range from the box. Colours correspond to the treatments: sorghum genotypes CK60B (grey), Ochuti (orange), Tiemarifing (purple); nutrient treatments: Base (grey), low level of micro- and macronutrients; +Micro (green), base level supplemented with micronutrients; +Macro (blue), base level supplemented with macronutrients; +MicroMacro (red), optimal level of micro- and macronutrients.

There was no significant interaction between genotype and nutrient treatments for ASNPC and total striga biomass (Table 3). Sorghum genotypes had a significant (*P* = 0.016) effect on total striga biomass but not on ASNPC (Table 3; Fig. 1A). Striga biomass was significantly higher in CK60B than in Ochuti and Tiemarifing (*P* < 0.05; Supplementary Data Fig. S1). Nutrients had a highly significant (*P* < 0.001) effect on both cumulative striga numbers (ASNPC) and total striga biomass (Table 3). Macronutrient treatments (+Macro, +MicroMacro) showed significant lower striga infection levels (both ASNPC and striga biomass) compared to the other nutrient treatments (Base, +Micro). The treatments supplemented with macronutrients (+Macro and +MicroMacro) were never significantly different from one another (Fig. 1B).

**Table 3.** ANOVA output of the mixed effects model of the effect of sorghum genotypes [G] and nutrient treatments [N] on area under the striga number progress curve (ASNPC) and total striga biomass. Bold type indicates significant differences at *P* < 0.05.

Source of variation	d.f.	ASNPC		Total striga biomass	
		*F*-value	*P*-value	*F*-value	*P*-value
Genotype [G]	2	1.8	0.192	5.1	**0.016**
Nutrients [N]	3	33.9	**<0.001**	38.6	**<0.001**
G × N	6	1.8	0.113	0.4	0.871

d.f. = degrees of freedom.

### Leaf-level host plant performance

There was no significant genotype by nutrient interaction effect on any leaf-level indicator (CO_2_ assimilation, stomatal conductance and electron transport rate, chlorophyl content) of both striga-free and striga-infected host plants at 60 DAS (Table 4; Supplementary Data Table S1). The effects of the treatments on CO_2_ assimilation, stomatal conductance and electron transport rates were comparable. Thus, we only present here the results for CO_2_ assimilation and leaf chlorophyll content. The results for stomatal conductance and electron transport are presented in Figs S2 and S3.

**Table 4.** ANOVA output of the mixed-effects model of the effect of sorghum genotypes [G] and nutrient treatments [N] on sorghum CO_2_ assimilation rate and leaf chlorophyll content at 60 DAS. Bold type indicates significant differences at *P* < 0.05.

	Source of variation	d.f.	*Striga*-free plants		*Striga*-infected plants	
			*F*-value	*P*-value	*F*-value	*P*-value
CO_2_ assimilation rate	Genotype [G]	2	0.1	0.899	52.1	**0.0001**
	Nutrient [N]	3	0.5	0.685	11.0	**0.0001**
	G × N	6	0.5	0.832	0.7	0.69
Leaf chlorophyll content	Genotype [G]	2	3.1	0.066	13.2	**0.0002**
	Nutrient [N]	3	13.9	**0.0001**	6.1	**0.0008**
	G × N	6	1.6	0.1486	1.5	0.179

d.f. = degrees of freedom.

**Fig. 2.**
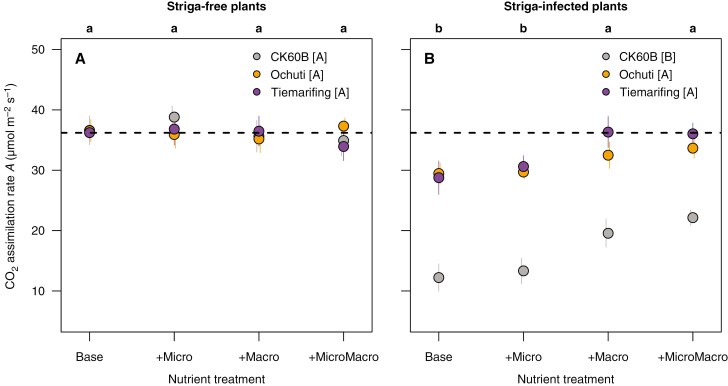
Effect of sorghum genotype and nutrient treatments on CO_2_ assimilation rate (*A*, μmol m^−2^ s^−1^) of striga-free plants (A) and striga-infected plants (B) at 60 DAS. Points show the mean of the data for the 12 combinations of genotype and nutrient treatment levels across the three experiments. Error bars represent ±1× the standard error of the original data. Lower-case letters on top of each graph indicate significant differences among the nutrient treatments at *P* < 0.05 based on comparison of estimated marginal means across the three experiments. Upper-case letters within the key indicate significant differences among sorghum genotypes at *P* < 0.05 based on comparison of estimated marginal means across the three experiments. Horizontal dashed lines indicate the mean photosynthesis rate of striga-free plants across genotypes and nutrient treatments, as a reference. The colour of the points corresponds to the sorghum genotype: CK60B (grey), Ochuti (orange) and Tiemarifing (purple).

**Fig. 3.**
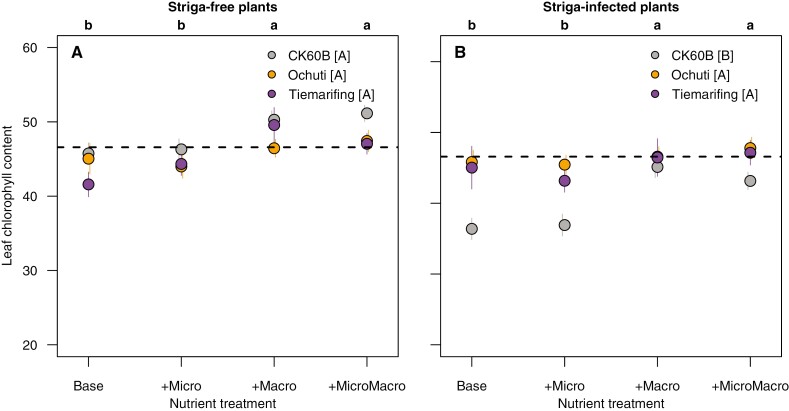
Effect of sorghum genotype and nutrient treatments on leaf chlorophyll content of striga-free plants (A) and striga-infected plants (B) at 60 DAS. Points show the mean of the data for the 12 combinations of genotype and nutrient treatment levels across the three experiments. Error bars represent ±1× the standard error of the original data. Lower-case letters on top of each graph indicate significant differences among the nutrient treatments at *P* < 0.05 based on comparison of estimated marginal means across the three experiments. Upper-case letters within the key indicate significant differences among sorghum genotypes at *P* < 0.05 based on comparison of estimated marginal means across the three experiments. Horizontal dashed lines indicate the mean leaf chlorophyll content of striga-free plants across genotypes and nutrient treatments, as reference. The colour of the points corresponds to the sorghum genotype: CK60B (grey), Ochuti (orange) and Tiemarifing (purple).

Under striga-free conditions, there were no significant genotype or nutrient treatment effects on leaf CO_2_ assimilation rate and only significant nutrient effects on leaf chlorophyl content (Table 4). Under striga-infested conditions, significant sorghum and nutrient treatments effects were observed on both CO_2_ assimilation rate and leaf chlorophyll content (Table 4). Among the genotypes, striga-infected Ochuti and Tiemarifing plants always showed significantly higher values of both leaf-level indicators than CK60B (Figs 2 and 3). Macronutrient treatments (+Macro, +MicroMacro) showed significantly higher CO_2_ assimilation and leaf chlorophyll content compared to the other nutrient treatments (Base, +Micro) in striga-infected plants (Figs 2 and 3). The nutrient treatments supplemented with macronutrients (+Macro and +MicroMacro) were never significantly different from one another.

### Overall host plant performance

**Fig. 4.**
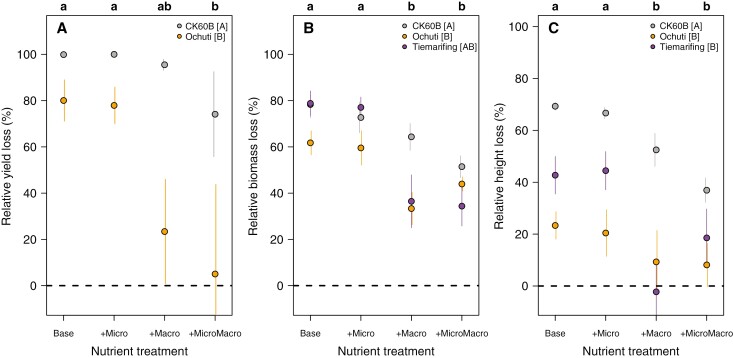
Effect of sorghum genotypes and nutrient treatments on the relative losses of yield production (A), total biomass (B) and height at maturity [85 DAS] (C). Points show the mean of the data for the 12 combinations of genotype and nutrient treatment levels across the three experiments. Error bars represent ±1× the standard error of the original data. Lower-case letters on top of each graph indicate significant differences among the nutrient treatments at *P* < 0.05 based on comparison of estimated marginal means across the three experiments. Upper-case letters within the key indicate significant differences among sorghum genotypes at *P* < 0.05 based on comparison of estimated marginal means across the three experiments. Horizontal dotted line indicates no relative loss, as reference. The colour of the points corresponds to the sorghum genotype: CK60B (grey), Ochuti (orange) and Tiemarifing (purple).

**Fig. 5.**
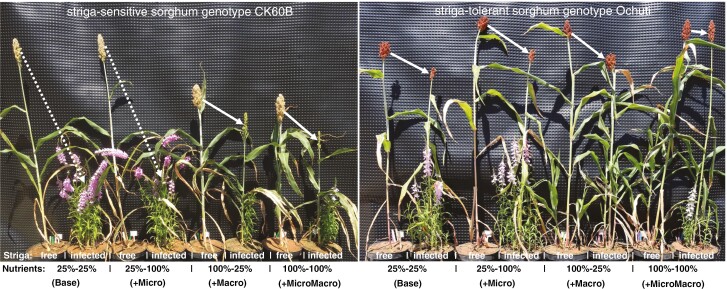
Phenotypes of a striga-sensitive sorghum genotype, CK60B (left), and a striga-tolerant genotype, Ochuti (right), subjected to four nutrient treatments. Striga-free and striga-infected plants are paired within each nutrient treatment level. White arrows show differences in height and panicle sizes across the treatments.

Both genotypes and nutrient treatments significantly impacted relative sorghum grain yield losses (Table 5). Across nutrient treatments, CK60B showed a significantly higher relative yield loss than Ochuti. Treatments with macronutrients (+Macro, +MicroMacro) significantly reduced grain yield losses. For the tolerant genotype Ochuti, the +MicroMacro treatment completely restored yield, whereas for sensitive CK60B yield losses at this nutrient treatment level remained at 74 % (Figs 4A and 5).

**Table 5.** ANOVA output of the mixed effects model of the effect of sorghum genotypes [G] and nutrient treatments [N] on relative losses of yield, dry biomass and height at 85 DAS. Bold type indicates significant differences at *P* < 0.05.

Source of variation	d.f.	Relative yield loss		d.f.	Relative biomass loss		Relative height loss	
		*F*-value	*P*-value		*F*-value	*P*-value	*F*-value	*P*-value
Genotype [G]	1	17.0	**0.002**	2	7.8	**0.003**	31.5	**0.001**
Nutrient [N]	3	5.4	**0.002**	3	19.5	**<0.001**	11.1	**0.001**
G × N	3	1.9	0.147	6	2.2	0.052	1.9	0.12

d.f. = degrees of freedom.

There was a marginally significant (*P* < 0.052) genotype by nutrient interaction effect and strong main effects on relative sorghum biomass loss (Table 5). The +Macro treatment had a stronger loss reduction effect on the two tolerant genotypes (Tiemarifing and Ochuti) than on the sensitive genotype (CK60B) (Fig. 4B). Overall, highest relative biomass losses were observed with CK60B and lowest losses were observed with Ochuti. The low nutrient (Base) and micronutrient treatment (+Micro) resulted in the highest relative biomass losses while treatments with macronutrients (+Macro, +MicroMacro) significantly reduced these losses (Fig. 4B).

There were also significant genotype and nutrient effects on relative height loss at 85 DAS (Table 5). CK60B showed significantly higher losses in height than Ochuti and Tiemarifing, which showed comparable relative height loss (Fig. 4C). The low nutrient (Base) and micronutrient (+Micro) treatments showed the highest height losses compared to the treatments with macronutrients (+Macro and +MicroMacro). The application of macronutrients (+Macro) restored sorghum plant height only when applied to the tolerant genotypes (Tiemarifing and Ochuti) (Fig. 4C).

## DISCUSSION

Combining improved host plant nutrition with host plant defence is likely to result in better control than relying solely on one approach, as previously proposed by Mwangangi *et al.* (2021). We evaluated how different nutrient levels and composition influence striga infection and host performance of genotypes differing in striga tolerance.

### Genotype and nutrient effects on striga infection

The inherent differences in striga resistance across genotypes, causing variations in striga infection levels, poses a challenge to accurately assess striga tolerance within an experiment (Rodenburg *et al.*, 2005, 2006). To make a fair assessment of striga effects on host plants across different genotypes, it is necessary to ensure similar baseline infection levels (Rodenburg and Bastiaans, 2011). This was targeted in this study, and across the three sorghum genotypes, CK60B, Ochuti and Tiemarifing, a similar striga infection level was achieved. This confirmed the fair and accurate selection of the sorghum genotypes for this study. While striga infection numbers were similar, differences were observed in striga biomass across the genotypes. CK60B, a sensitive genotype, showed a significantly higher striga biomass than the tolerant genotypes, Ochuti and Tiemarifing. These differences correspond with previously reported differences between sensitive and tolerant cultivars (van Ast *et al.*, 2000; van Ast and Bastiaans, 2006). In the current study, it appears that if the parasite has a stronger effect on a host plant because the genotype is sensitive (CK60B) it is also better able to extract resources from it than from a tolerant host plant genotype (Ochuti or Tiemarifing). Hence differences in parasitic biomass acquisition may define differences in tolerance levels of the host plant genotype, rather than resistance. Improved nutrient supply of macro- and micronutrients reduced both striga infection numbers and striga biomass. Supplying high levels of nitrogen and phosphorus potentially reduces strigolactone production by host plants, which, in turn, leads to a reduction in striga germination and, consequently, a decrease in striga numbers, as demonstrated by Jamil *et al.* (2013). Additionally, nitrogen supply impairs the formation of the striga root penetration structure, the haustorium, resulting in failed parasitism (Kokla *et al.*, 2022). Improved supply of nutrients has also been shown to reduce striga infection levels after germination and haustorium formation (Mwangangi *et al.*, 2023), but the mechanism is not yet well understood.

### Genotype and nutrient effects on striga-induced host plant damage

Striga tolerance, defined as the ability of a host plant to withstand striga infection by exhibiting comparatively lower striga-induced damage, is an essential component of integrated striga management (Rodenburg and Bastiaans, 2011) and genetic variation in striga tolerance is observed across rice and sorghum genotypes (Rodenburg *et al.*, 2005, 2006, 2017). Previous studies have also shown that applying fertilizers, specifically nitrogen, can improve the performance of striga-infected host plants under controlled conditions (Press and Stewart, 1987) as well as in the field (Boukar *et al.*, 1996; Showemimo *et al.*, 2002; Kamara *et al.*, 2007; Gebremariam and Assefa, 2015). However, these studies failed to demonstrate whether this advantage was derived from an increase in overall host plant performance, an increase in host plant tolerance, a reduced striga infection level or a combination of these effects. Moreover, previous studies focused primarily on the effects of nitrogen and phosphorus fertilizers (Kamara *et al.*, 2007; Gebremariam and Assefa, 2015; Tippe *et al.*, 2017) and little is known about the effects of other essential nutrients.

Accurately studying striga tolerance requires: (1) striga-infested next to striga-free host plants, (2) a known striga-sensitive next to a known striga-tolerant host plant genotype and (3) similar parasite infection loads across the genotypes. In the current study these preconditions were met, which paves the way for answering the following research questions: (1) to what extent does micro- or macronutrient supply play a role in mitigating striga effects on host plant performance? and (2) does the extent of reduction of striga-induced losses by improved nutrition depend on the physiological tolerance level of the host plant?

#### To what extent does micro- or macronutrient supply play a role in mitigating striga effects on host plant performance?

The adverse effects of striga infection caused host plant losses at both leaf and plant level. The level and type of nutrient supply showed variation in reducing striga-induced losses. Phytopathology studies have shown that improving the nutritional status of host plants has a positive impact on plant health, vigour and immunity (Dordas, 2009; Tripathi *et al.*, 2022). In studies of parasitic weeds, nitrogen fertilizers have been shown to improve yield and biomass of striga-infected sorghum plants (Showemimo *et al.*, 2002; Gebremariam and Assefa, 2015; Gebremedhin *et al.*, 2021). In the current study, low macronutrient treatments (Base or +Micro) led to the highest relative losses across multiple parameters: 91–92 % for yield, 71–74 % for biomass and 45–46 % for height. Application of macronutrients resulted in lower losses: 41–60 % for yield, 44–45 % for biomass and only 21–22 % for height, respectively.

Consistent with the findings at the whole plant level, improved nutrient supply showed comparable effects at the leaf level. The low macronutrient treatments resulted in 24–35 % losses across the gas exchange variables while treatments with increased macronutrients showed only 2–14 % losses across genotypes. The mechanistic background of how striga suppresses host photosynthesis is still not well understood. However, studies have shown that striga infection increases host plant levels of ABA, which causes a decrease in host-leaf stomatal conductance and hence a reduction in the host transpiration rate (Frost *et al.*, 1997; Taylor and Seel, 1998; Fujioka *et al.*, 2019) and photosynthesis levels (Press and Stewart, 1987; Press *et al.*, 1987). From the current study, we hypothesize that application of macronutrients (+Macro, +MicroMacro) improved photosynthesis rates by suppressing striga-induced increases of host ABA levels. Applying phosphorus, potassium and nitrogen indeed decreases ABA levels in plants (Battal, 2004; Fang *et al.*, 2018; De Souza Osório *et al.*, 2020). Moreover, nitrogen fertilizer application was shown to increase the transpiration rate of striga-infected pearl millet, indicating increased stomatal conductance and photosynthesis (Boukar *et al.*, 1996). Various studies have shown the importance of macronutrients in leaf gas-exchange functions. The application of nitrogen (Gworgwor and Weber, 1991, Song *et al.*, 2019), calcium (Hu *et al.*, 2018; Naeem *et al.*, 2018) and potassium (Wei *et al.*, 2013; Zain *et al.*, 2014) enhances photosynthesis and gas exchange during drought stress (Press *et al.*, 1987; Gworgwor and Weber, 1991; Boukar *et al.*, 1996; Song *et al.*, 2019). Furthermore, increased chlorophyll content has been consistently observed following the application of nitrogen, potassium and calcium during drought stress (Nayek *et al.*, 1983; Taran *et al.*, 2017; Abdel-Motagally and El-Zohri, 2018; Agami *et al.*, 2018; Dimkpa *et al.*, 2020). In the current study, both treatments with supplemented macronutrients significantly increased leaf chlorophyll content, which could further explain improvements in host plant photosynthesis.

#### Does the extent of reduction of striga-induced losses by improved nutrition depend on the physiological tolerance level of the host plant?

Macronutrient applications reduced the adverse effects of striga on the host plant at the leaf and plant level. However, the degree of reduction of the striga-induced losses depended on the physiological tolerance level of the host plant. While indications of this were reported previously from field studies by Showemimo *et al.* (2002) and Tippe *et al.* (2020), the current study is the first to quantifiably demonstrate this using an adequate experimental set-up. Here we showed that improved host plant nutrition was insufficient to completely alleviate striga damage in a sensitive sorghum genotype (CK60B), whereas supplying macronutrient treatments to tolerant genotypes (Ochuti and Tiemarifing) completely restored leaf-level physiological performance of the host plant. The treatment effects on host plant stem height, (above-ground) biomass and grain yield losses are similar to what we observed at the leaf level. Striga-induced grain losses of 99.9 % for CK60B were mitigated to 74 % through macro- and micronutrient availability. In the case of Ochuti, a tolerant genotype, the grain losses of 80 % were reduced to a mere 5 % in the presence of macro- and micronutrient treatments. Host plants with higher physiological tolerance to striga indeed respond more positively to improved nutrition as previously postulated by Mwangangi *et al.* (2021).

The strong effects we observed on yield are presumably the result of a multiplication effect of combining increased nutrient availability and host physiological tolerance to striga. We need to stress that the striga infestation levels used in our pot experiments were much higher than common in field-level studies (see, for instance, Rodenburg *et al.*, 2005). Therefore, we are confident that the combination of tolerance and improved nutrient supply will provide effective striga control under field conditions.

### Implications for striga control and recommendations for further research

Neither tolerant host plant genotypes nor improved nutrition applied alone completely cancels out the negative effects of striga infection on sorghum growth and production. Tippe *et al.* (2020) showed that the application of NPK fertilizer (nitrogen, phosphorus, potassium) improved rice yields in striga-infested field conditions but did not completely offset the striga-induced host damage. Furthermore, Rodenburg *et al.* (2005, 2006) showed that tolerant genotypes showed lower relative yield losses than sensitive genotypes, but striga effects were still notable. The current study showed that when macronutrients are combined with a sensitive sorghum genotype, excessively high yield losses because of striga infection may still be expected. However, if these nutrients are applied to tolerant genotypes, the yield of striga-infected host plants may be almost restored to the same level as striga-free host plants. Therefore, in terms of field-level applications, macronutrient-based fertilizers combined with tolerant genotypes comprise a feasible and promising method to avoid crop losses in striga-infested areas.

The findings of this study are based on a composite of macronutrients and a composite of micronutrient fertilizers. Future research should zoom in to study the effects of individual or combined elements to improve and simplify fertilizer compositions to make them more efficient and cost-effective. Also, studies on dose–effect relationships should be done, to assess the optimal dosage and timing of fertilizer application that should be applied for good striga control/crop performance at affordable input costs. This would be a helpful innovation for smallholder farmers for whom mineral fertilizers are often too expensive (Emechebbe *et al.*, 2004; Misiko *et al.*, 2011; Tippe *et al.*, 2017). Finally, there is a need to fine-tune fertilizer application methods. The application of nutrients directly to the host plant foliage might potentially be a more effective delivery route in terms of striga damage mitigation as proposed by Mwangangi *et al.* (2021).

Pending confirmation from the field, the current study implies that if farmers in striga-infested fields combine tolerant cultivars and macronutrient fertilizers, they may maintain crop productivity at levels almost comparable to that obtained under striga-free conditions.

## SUPPLEMENTARY DATA

Supplementary data are available at *Annals of Botany* online and consist of the following.

Table S1: ANOVA output of the mixed-effects model of the effect of sorghum genotypes [G] and nutrient treatments [N] on sorghum stomatal conductance rate and electron transport rate at 60 DAS. Figure S1: Sorghum genotype (A) and nutrient (B) treatments on total biomass dry weight of striga plants at harvest. Figure S2: Effect of sorghum genotype and nutrient treatments on stomatal conductance rate (GSW, mol m^−2^ s^−1^) on striga-free plants (A) and striga-infected plants (B) at 60 DAS. Figure S3: Effect of sorghum genotype and nutrient treatments on electron transport rate (ETR, μmol^2^ s^−1^) in striga-free plants (A) and striga-infected plants (B) at 60 DAS.

mcae031_suppl_Supplementary_Figures_S1

mcae031_suppl_Supplementary_Figures_S2

mcae031_suppl_Supplementary_Figures_S3

mcae031_suppl_Supplementary_Tables_S1
