# Supplementary figures and images for: Macronutrient application rescues performance of tolerant sorghum genotypes when infected by the parasitic plant striga
Source: Ann Bot. 2024 Mar 1;134(1):59–70. doi: 10.1093/aob/mcae031 (PMC11161562; doi:10.1093/aob/mcae031)

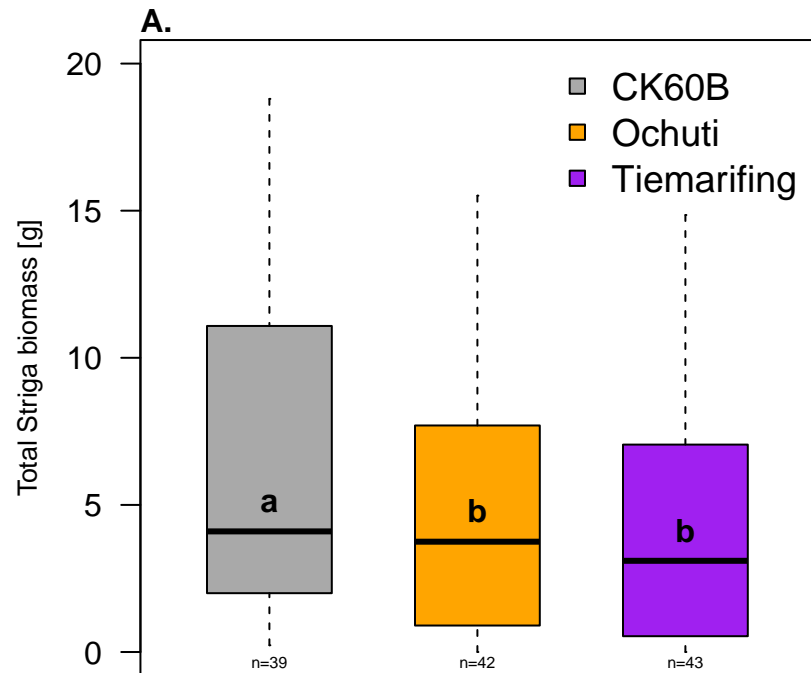

Genotype treatment

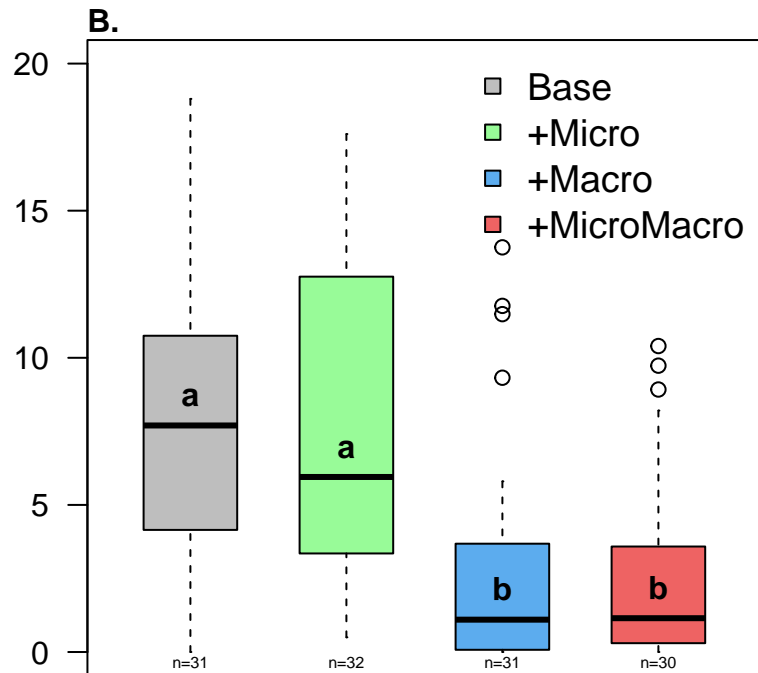

Nutrient treatment

Supplement: mcae031_suppl_Supplementary_Figures_S1 [file mcae031_suppl_supplementary_figures_s1.pdf]

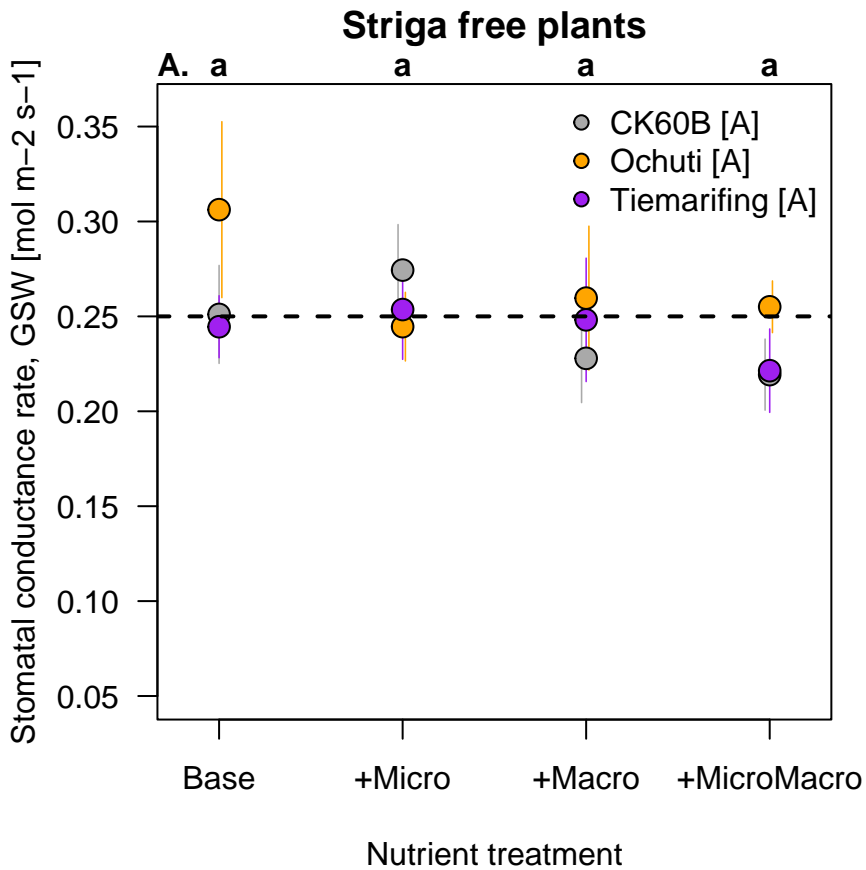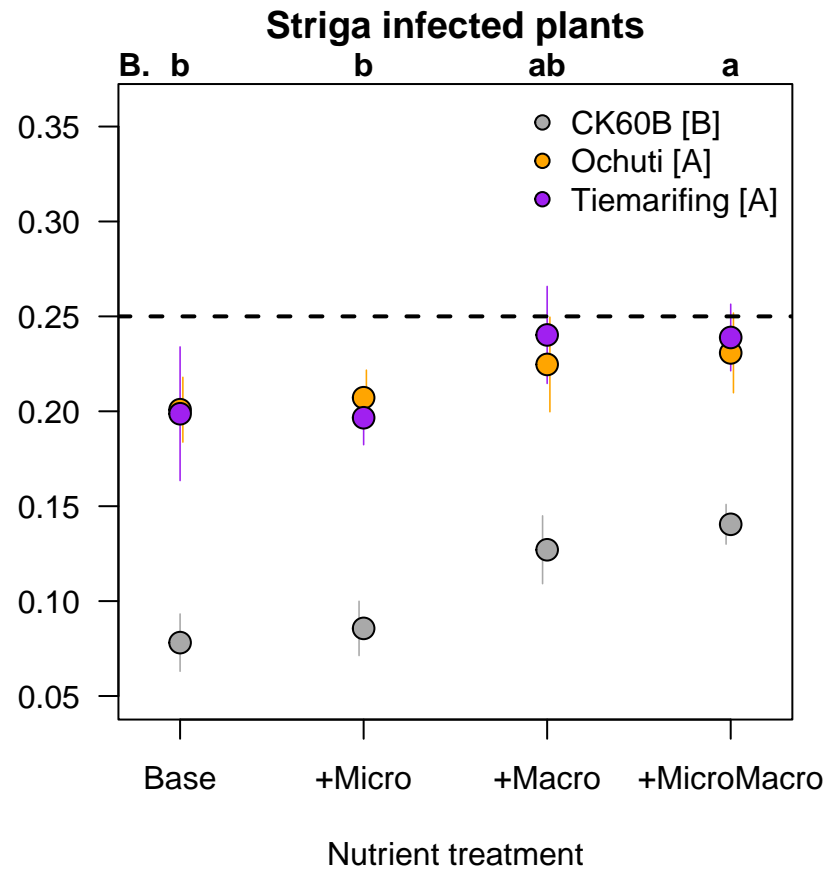

Supplement: mcae031_suppl_Supplementary_Figures_S2 [file mcae031_suppl_supplementary_figures_s2.pdf]

Electron transport rate, ETR [ $\mu\text{mol s}^{-1}$ ]

## Striga free plants

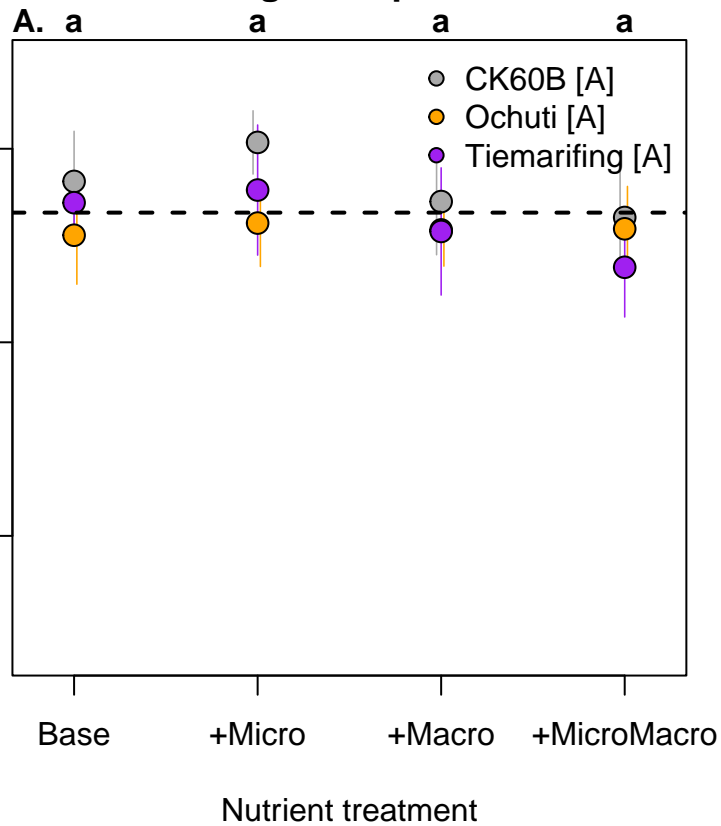

## Striga infected plants

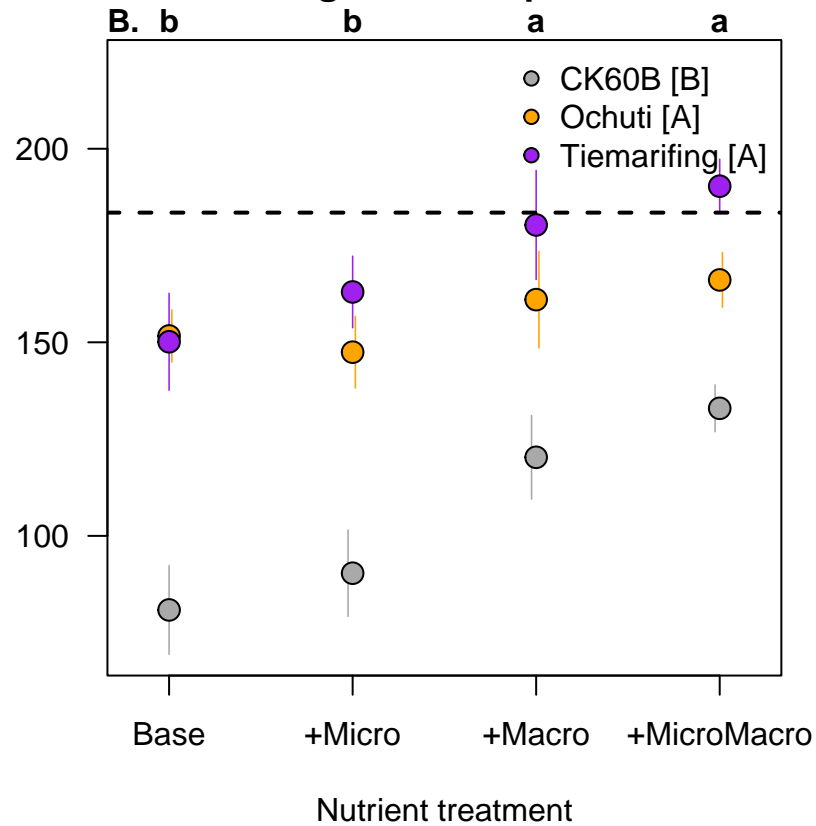

Supplement: mcae031_suppl_Supplementary_Figures_S3 [file mcae031_suppl_supplementary_figures_s3.pdf]
