# Supplementary material for: Macronutrient application rescues performance of tolerant sorghum genotypes when infected by the parasitic plant striga
Source: Ann Bot. 2024 Mar 1;134(1):59–70. doi: 10.1093/aob/mcae031 (PMC11161562; doi:10.1093/aob/mcae031)
Supplement: mcae031_suppl_Supplementary_Tables_S1 [file mcae031_suppl_supplementary_tables_s1.docx]

**Supplementary Data**

**Table S1: ANOVA output of the mixed-effects model of the effect of sorghum genotypes [G] and nutrient treatments [N] on sorghum stomatal conductance rate and electron transport rate at 60 DAS.** The highlighted numbers show significant differences at p < 0.05.

|  |  |  | **Striga free plants** | | **Striga infected plants** | |
| --- | --- | --- | --- | --- | --- | --- |
|  | Source of variation | df | F-value | p-value | F-value | p-value |
| Stomatal conductance rate | Genotype [G] | 2 | 0.9 | 0.438 | 27.5 | <0.0001 |
|  | Nutrient [N] | 3 | 1.2 | 0.299 | 4.6 | 0.005 |
|  | G * N | 6 | 0.8 | 0.607 | 0.2 | 0.965 |
| Electron transport rate | Genotype [G] | 2 | 1.2 | 0.32 | 29.8 | <0.0001 |
|  | Nutrient [N] | 3 | 0.8 | 0.484 | 11.1 | <0.0001 |
|  | G * N | 6 | 0.2 | 0.974 | 1.1 | 0.391 |

df = degrees of freedom
